# Efficient Molecular Rectification in Metal–Molecules–Semimetal Junctions

**DOI:** 10.1021/acs.jpclett.4c02900

**Published:** 2024-10-15

**Authors:** Shachar Shmueli, Mor Cohen Jungerman, Pini Shekhter, Yoram Selzer

**Affiliations:** † School of Chemistry, Tel Aviv University, Tel Aviv 69978, Israel; ‡ The Tel Aviv Center for Nanoscience and Nanotechnology, Tel Aviv 69978, Israel

## Abstract

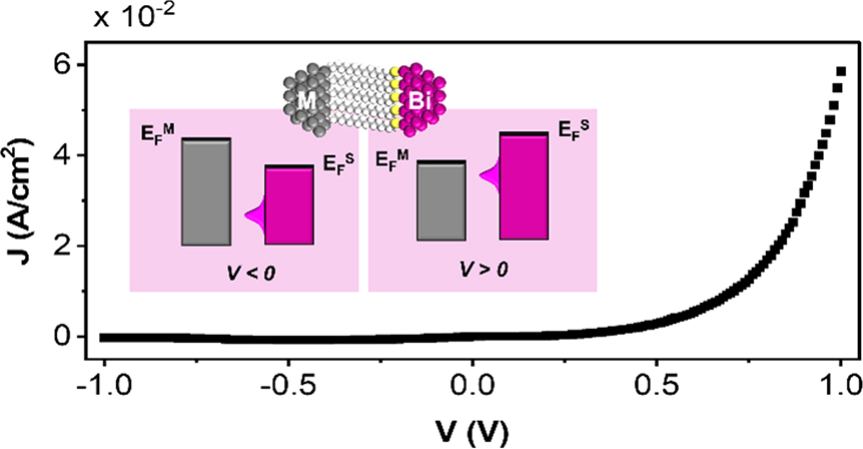

Molecular rectification is expected to be observed in metal–molecule–metal tunnel junctions in which the resonance levels responsible for their transport properties are spatially localized asymmetrically with respect to the leads. Yet, effects such as electrostatic screening and formation of metal induced gap states reduce the magnitude of rectification that can be realized in such junctions. Here we suggest that junctions of the form metal–molecule(s)–semimetal mitigate these interfacial effects. We report current rectification in junctions based on the semimetal bismuth (Bi) with high rectification ratios (>10^2^) at 1.0 V using alkanethiols, molecules for which rectification has never been observed. In addition to the alleviation of screening and surface states, the efficient rectification is argued to be related to symmetry breaking of the applied bias in these junctions because of a built-in potential within the Bi lead. The significance of this built-in potential and its implications for the future and other applications are discussed.

Within the rich diversity of high-bias transport properties of molecular junctions,^1−6^ rectification is of unique importance, not just historically,^7,8^ but more importantly as a ’test case’ for the validity and reliability of molecular junctions as an alternative to silicon-based devices.^9^ Current rectification, apparent as an asymmetric I–V behavior of junctions as a function of bias polarity, is quantified by a rectification ratio: RR(V) = |I(V^+^)/I(V^–^)| for |*V*^+^| = |*V*^–^|. One of the most common mechanisms that has been suggested to either induce or interpret rectification in molecular junctions^10−46^ considers coherent transport through a spatially localized molecular level positioned asymmetrically between the two leads of a junction (Figure 1a). The corresponding transmission function is then described by a Breit–Wigner resonance expression:^23^
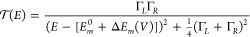
1where, *E*_*m*_^0^ is the energy position (relative to the Fermi level) of the localized molecular level at zero bias, Δ*E*_*m*_(*V*) is the bias-induced shift of this level and Γ_*L*,*R*_ are the coupling constants of the level to the left and right leads. Ignoring electrostatic screening effects,^47^ under an applied voltage, *V*, the potential drop on such a junction is assumed to be linear and hence divided between the two tunneling barriers in proportion to their lengths: *L*_*right*_ and *L*_*left*_. As a result, using η ≈ *L*_*right*_/*L*_*left*_, the energy shift of the level under such a bias is then: , where *e* is the charge of an electron (Figure 1b). The molecular level is aligned with the Fermi level of one of the leads when the applied (forward) potential is  and in reverse polarity when . If η ≠ 1 the junction is rectifying, and rectification increases as η diverges from this value (under the constrain that *L* = *L*_*left*_ + *L*_*right*_, where L is total length of the molecule).

**Figure 1:**
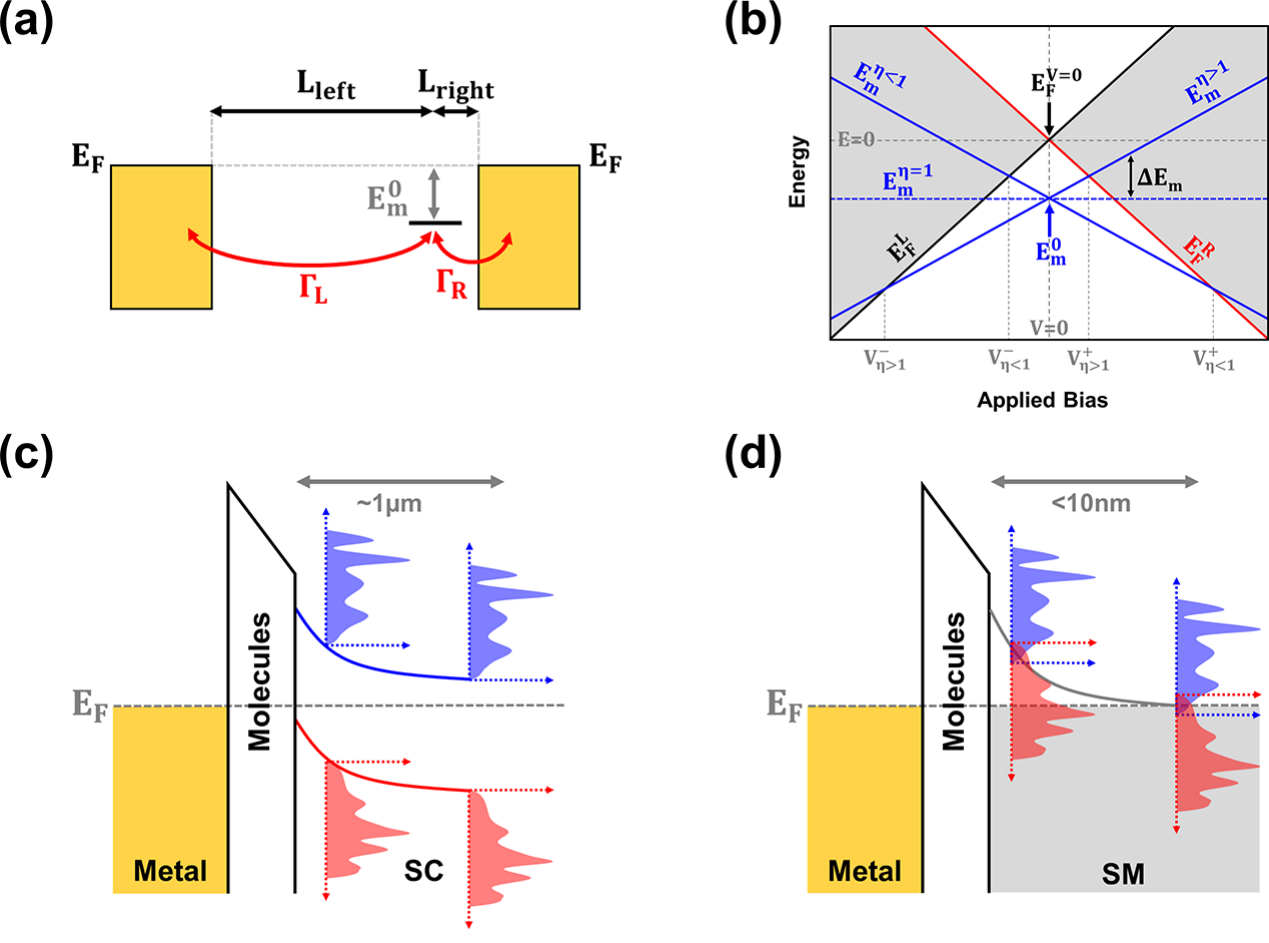
(a) A molecular level spatially located asymmetrically between two leads (*L*_*L*_ ≠ *L*_*R*_), with an energy *E*_*m*_^0^ below the Fermi level and coupled via tunneling barriers to both leads (see text for details). (b) The energy change (blue lines) of the resonance level under bias, Δ*E*_*m*_, for two different potential divisions: η>1 and η<1. The positive and negative bias values, *V*^±^, at which the molecular level enters the energy window between the two Fermi levels, E_F_, of the leads (shaded gray area between the red (*E*_*F*_^*R*^) and the black (*E*_*F*_^*L*^) lines), depend on η, i.e., the spatial location of the level. (c) A Schottky barrier in a metal-molecules-semiconductor (SC) junction. The DOS of the valence and conduction bands are depicted in red and blue, respectively. The built-in potential extends ∼1 μm into the semiconductor. (d) The barrier formed in a metal–molecules–semimetal (SM) junction. Unlike in (c), in this case the DOS at the Fermi level is not zero. In this case the built-in potential extends ∼10 nm into the semimetal.

Using this approach, experimental RR values in the order of 10^2^ are commonly reported, while based on eq 1, room temperature rectification, with small coupling to the leads and either η ≫ 1 or η ≪ 1 can be as high as RR ∼ 10^5^, which should make such devices technologically relevant.^9^ However, experiments reporting such high RR values, mainly in molecular ensemble junctions (MEJs), cannot fully explain rectification using the above mechanism and invoke additional processes such as changes in the molecular configuration or in the number of current-carrying molecules with bias^16,21^ to explain the observed rectification.

There are several reasons that cause experimental RR values to be lower than the theoretical values based on the above mechanism: (i) High (more than few meV) coupling values to the leads. As Γ increases, i.e., the resonance level becomes broader, the difference between the contributions of this level to the transmission at the two bias polarities becomes smaller and as a result RR decreases.^23,47^ (ii) The spatial extent of the localized electronic level. The theoretical limit assumes a point-like localized level. However, if the level is smeared along the molecule, it does not response uniformly to the applied bias and causes RR to decrease.^23^ (iii) Electrostatic screening within the molecule.^48^ As the screening length becomes shorter, the potential distribution across the molecule deviates more from linearity and the energy shift Δ*E*_*m*_ for a given *V*, becomes smaller (η effectively becomes more equal to 1). (iv) Metal induced gap states (MIGS) and Fermi level pinning,^36,41,49,50^ both also cause Δ*E*_*m*_ to behave not according to the assumed linear potential divider across the junction.

Elimination of the above causes or deterministic control of their magnitudes at the molecular level is very difficult and beyond current capability. Thus, full harnessing of the RR ∼ 10^5^ that can be achieved by this model necessitates an additional mechanism that can better break symmetry in junctions under bias and enable Δ*E*_*m*_(*V*) to behave more asymmetrically with bias polarity. We suggest that such a mechanism can be realized if a semimetal is used as one of the leads in such junctions. To understand the suggested approach, consider Figures 1c and 1d. When a Schottky barrier is formed between a metal and a semiconductor (Figure 1c), the resulting current rectification is due to both the formed built-in potential within the semiconductor and the zero density of states (DOS) at the Fermi level within its energy gap. In contrast, when a junction between a metal and a semimetal is established (Figure 1d), a built-in potential that causes a shift of electronic levels is also formed, however in this case the DOS at the Fermi level is never zero because semimetals lack an energy gap between their electronic bands at this level. Thus, when a potential is applied on junctions of the form metal-molecules-semimetal, rectification is not achieved as in Schottky barriers, instead it is formed because the applied bias in such junctions is divided between the molecules and within the semimetal. As this division is not symmetric with bias polarity (will be shown blow), it induces rectification by changing the way in which Δ*E*_*m*_ responses to the applied bias.

We have recently demonstrated that indeed a built-in potential is formed within the semimetal side of such junctions using junctions of the form metal-alkanethiols-bismuth (Bi).^51^ The important properties of Bi that make it suitable for the purpose of this study are its low density of charge carriers: ∼ 10^17^ cm^–3^ (10^22^ cm^–3^) and its low density of states (DOS) at the Fermi level: ∼ 4.2 × 10^–6^ states eV^–1^ atom^–1^ (∼0.1 states eV^–1^ atom^–1^). The numbers in brackets are, for comparison, the values in Au.^52^ Using thermovoltage measurements we have shown that the formed built-in potential within the Bi causes a shift of its electronic levels which also causes a change in the renormalization of the molecular levels. As a result, while for alkanethiols the electronic structure is independent of length, when attached to Bi as one of the leads, *E*_*m*_^0^ becomes length dependent.^51^ The change in *E*_*m*_^0^ as a function of molecular length was shown to be quantitatively correlated with the change induced by each of these molecules to the work function of Bi which in turn governs the magnitude of the built-in potential within the Bi.^51^

The same measurements also proved that since the DOS at the Fermi level of Bi is low, junctions with Bi leads are devoid of MIGS to the extent that their thermoelectric characteristics are not masked by these states but instead become fully dictated by the properties of their molecular part. This allowed us to show, for the first time and in accordance with theory, that the Seebeck coefficient of junctions conducting by a superexchange tunneling mechanism indeed increases with molecular length.^51^

To demonstrate the advantage of a semimetal lead to improve rectification it would be advantageous to use molecules that on one hand are expected to be highly rectifying and yet on the other hand have never been successfully used for this purpose due to the reasons discussed above. This makes alkanethiols highly appropriate because of the following reasons: (i) Their highest occupied molecular orbital (HOMO) is localized on their thiol end group, and therefore these molecules are expected to deliver efficient rectification^41^ with RR > 10^2^. (ii) Since in these molecules, because of the location of the HOMO, the potential divider ratio, η, deviates substantially from 1, based on the above expressions for *V*^±^, the value of RR is expected to saturate with increasing molecular length. If on the other hand such a behavior is not observed, it would imply that a different mechanism is responsible for the asymmetric response of the potential drop to bias polarity. (iii) So far, no significant rectification has been observed in junctions with alkanethiols assembled on typical metal leads. This has been attributed to negligible Δ*E*_*m*_ values due to Fermi level pinning and MIGS. Thus, observed rectification with these molecules assembled on Bi, would be another demonstration of the advantage of exploiting the low DOS in this semimetal.^53,54^

As in our previous study the junctions are based on self-assembled monolayers (SAMs) of alkanethiols (C_n_H_2n+1_SH) with n = 10, 12, 14, 16, 18 (abbreviated as SC_10_, SC_12_, SC_14_, SC_16_ and SC_18_), all assembled on 60 nm thick template-stripped Bi^TS^ films. Details of the characterization of the layers, type of bonding at the interface and proof for the elimination of native oxide can be found in the SI. An Eutectic Gallium–Indium (EGaIn) tip was used as a top soft contact in all measurements (Figure 2a). I–V curves were measured in the bias range of ±1.0 V, at room temperature under ambient conditions. The yield of stable junctions was >70%. The (area normalized) *I*-*V* curves for all junctions are plotted in Figure 2b, where *V* refers to the bias on the Bi film. The presented curves are an average of over 50 junctions, each measured 10 times. The data was collected from at least two different samples per chain length, with each junction measured at a different spot on the sample. The results in Figure 2b show chain-length dependent rectification with higher currents for positive bias values. The histograms in Figure 2c show that at a bias of 1.0 V, RR increases with chain-length with up to an average value of RR ∼ 10^2^ in the case of the longer chains SC_16_ and SC_18_. The dependence of RR on the applied bias is plotted in Figure 2d. These results are the first observation of rectification with alkanethiols and the RR values for the longer chains are at least an order of magnitude higher than many previously reported values for junctions based on alkanethiol chains connected at their end to various moieties with localized HOMO levels and attached via their thiols to typical metal leads (such as Au or Ag).^17,25−27,30,33,42^ Since here the HOMO is localized on the thiol, the polarity of the rectification is opposite to all previous studies.

**Figure 2:**
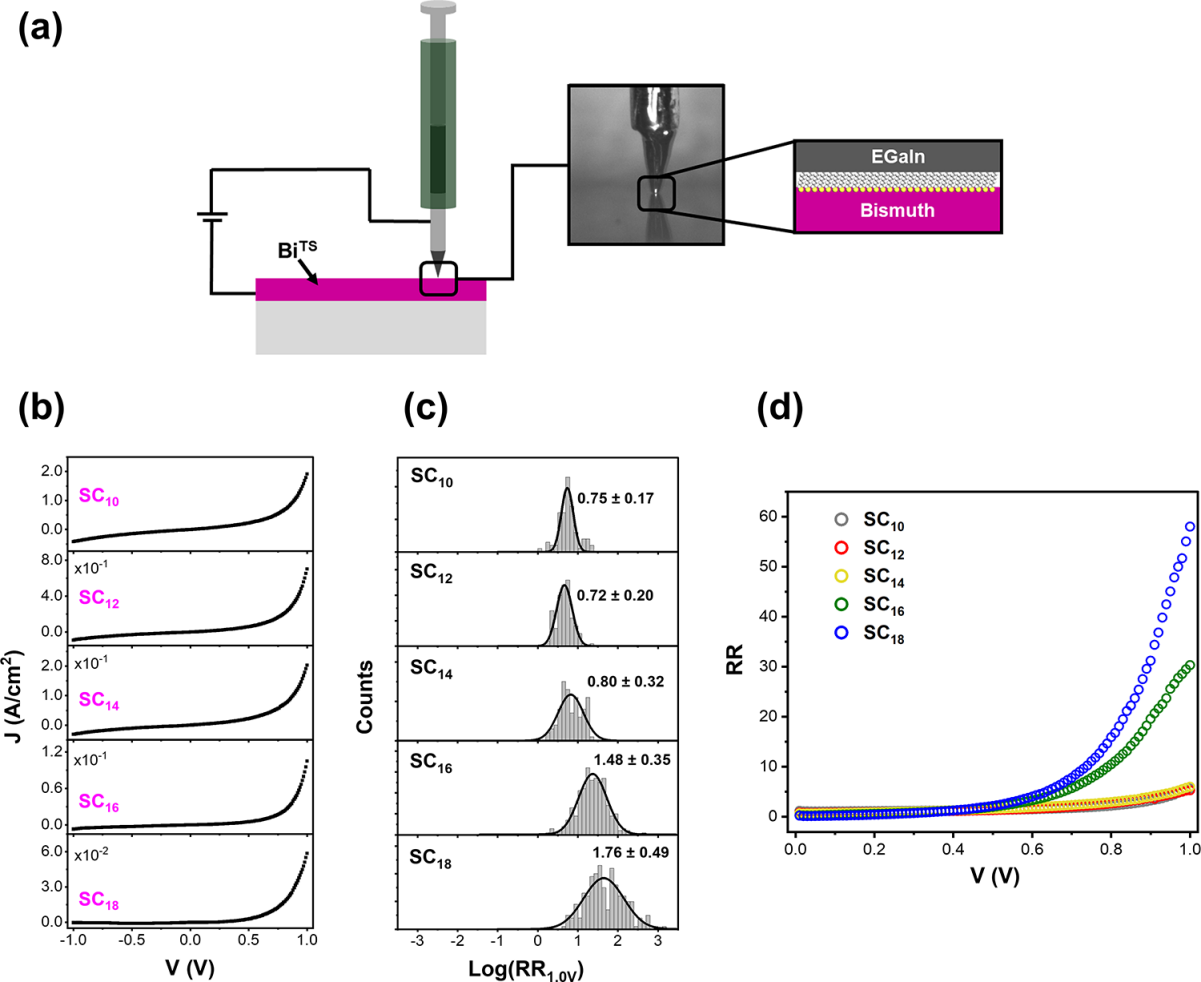
Experimental results and statistics. (a) A scheme of the experimental setup. (b) Averaged J-V curves for all SC_n_ chains. J is the area normalized current, calculated for each junction based on its observed cross section. The error of J is ∼10%. (c) Histograms of RR values at 1.0 V. The black lines present a log-normal fit, and the numbers are the mean values with their standard deviations. (d) Average RR values as a function of the applied bias for all chains.

Quantitative analysis of the results proceeds in four steps: (i) Calculation of *E*_*m*_^0^, for all chains, (ii) Calculation of the potential distribution across the molecules and within the Bi for each chain as a function of bias, (iii) Once the potential drop on the molecules is found, a calculation of the resulting shift of the resonance level Δ*E*_*m*_(*V*) can be made, and finally (iv) Calculation of the current through each junction at each bias using
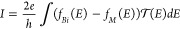
2where  is the transmission according to eq 1 and *f*_*Bi*_(*E*), *f*_*M*_(*E*) are the Fermi functions of the Bi and metal (EGaIn) leads, respectively. Details on each of these steps follow.

As discussed above, we have previously shown, using thermovoltage measurements, that the *E*_*m*_^0^ values of alkanethiols assembled on Bi within junctions are chain length dependent.^51^ In the SI we reproduce the calculation which transforms the change these monolayers induce to the work function of Bi, Δφ, (Figure 3a) into the built-in potential, *U*_0_ (Figure 3b), which in turn causes *E*_*m*_^0^ for each chain length to shift from its initial position. Thus, before any bias is applied: *E*_*m*_^0^ = −1.8*eV* + *U*_0_, where the initial position of the HOMO (identical for all chains) was determined by UPS.^51^

**Figure 3:**
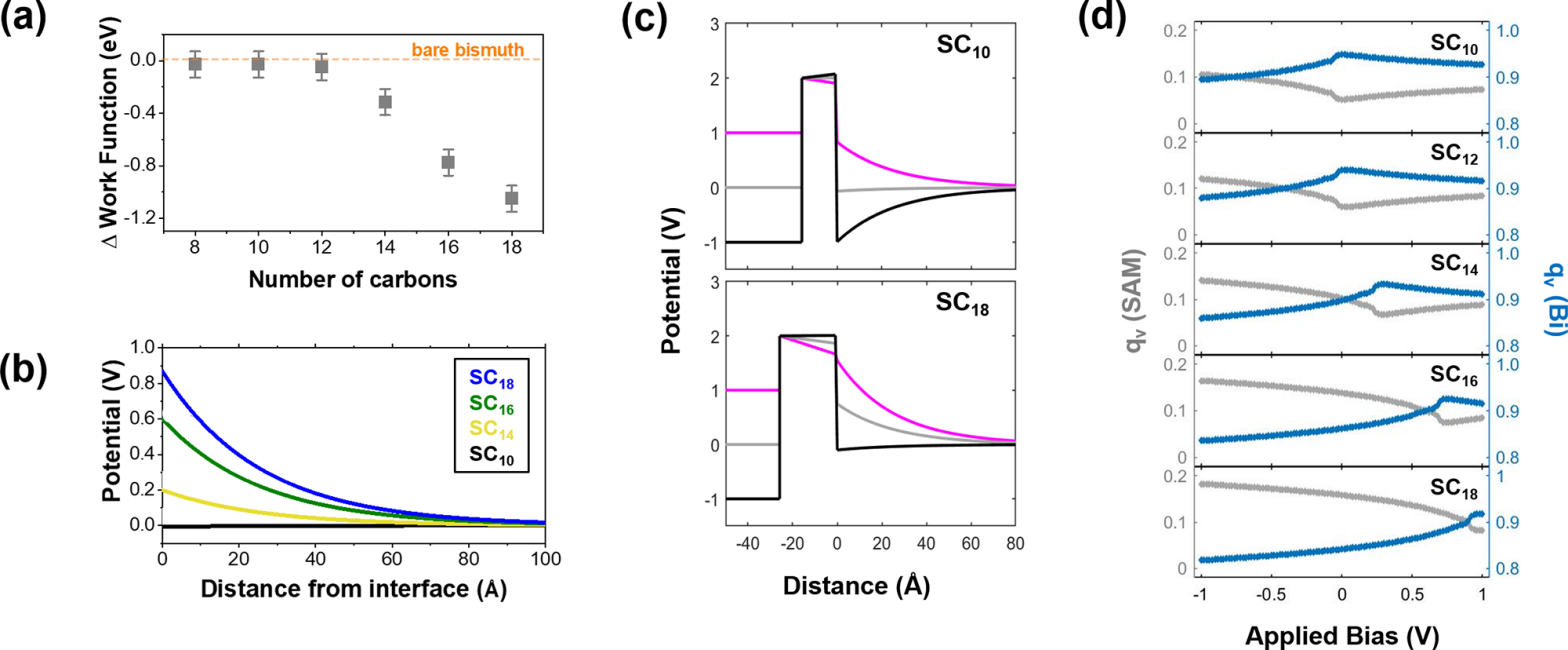
(a) The change in the work function of Bi with SAMS of alkanethiols. (b) The built-in potential formed within the Bi in GaIn–alkanethiol–Bi junctions. Note that the space-charge-region extends not more than ∼10 nm into the semimetal. (c) Calculated potential (ϕ) drops along SC_10_ and SC_18_ junctions for three bias values (gray: 0.0 V, black: + 1.0 V, magenta: −1.0 V). The calculation assumes linear drop on the SAM and exponential drop on the space-charge region in the Bi. (d) The fractions, q_v_(SAM) and q_v_(Bi) of the overall potential drop in the indicated junctions as a function of bias.

With a space charge region within the Bi, any applied voltage across a junction, *V*, also drops within the Bi side. A self-consistent calculation of the potential drop across the monolayers, ϕ_*SAM*_, needs also to account for *U*(*V*), i.e., how *U*_0_ is changing with bias. This calculation is detailed in the SI. Examples for the resulting spatial distribution of the potential across SC_10_ and SC_18_ junctions are plotted in Figure 3c for three *V* values. Since the value of Δφ with SC_10_ is small and causes the work function of Bi to be φ_*Bi*_ ∼ 4.27 eV, which is comparable to that of the EGaIn, φ_*metal*_ ∼ 4.3 eV, the resulting *U*_0_ is negligible and hence under bias |*U*(*V*^+^)| ≈ |*U*(*V*^–^)|. Most of the applied voltage in the SC_10_ junctions falls within the Bi lead. The variation of the potential across the SAM is small and correspondingly, Δ*E*_*m*_(*V*) is small and most importantly polarity symmetric. As a result, the rectification with SC_10_ is marginal. In contrast, since SC_18_ induces a large Δφ, the built-in potential within the Bi is large, *U*_0_ ∼ 0.9 eV, and *U*(*V*) behaves asymmetrically with bias polarity. As SC_18_ is 1 nm longer than SC_10_, the potential drop on the SAM, ϕ_*SAM*_, in junctions with the former molecule constitutes a larger fraction of *V*. Figure 3d shows how the fractions, *q*_*V*_(*SAM*) = ϕ_*SAM*_/*V* and *q*_*V*_(*Bi*) = *U*(*V*)/*V* of the applied potential on the SAMs and Bi, respectively, change with bias. In all junctions, for all bias values, *q*_*V*_(*SAM*) < *q*_*Bi*_(*SAM*). Since by definition: 1 = *q*_*V*_(*SAM*) + *q*_*V*_(*Bi*), any increase in *q*_*V*_(*SAM*) is accompanied by a decrease in *q*_*V*_(*Bi*). This plot essentially shows how rectification is induced since Δ*E*_*m*_(*V*) depends on *q*_*V*_(*SAM*). With increasing molecular length, the behavior of *q*_*V*_(*SAM*) becomes less symmetric around zero bias and consequently Δ*E*_*m*_(*V*^+^) ≠ Δ*E*_*m*_(*V*^–^). As the potential drop on the monolayer is *U*_0_ – *eV* – *U*(*V*), the shift Δ*E*_*m*_ is only a fraction of this value defined by the geometry of the potential divider. Because of the unique position of the localized (HOMO) resonance level in alkanethiols, instead of using the above ratio, η, it is more convenient to define the potential divider using the ratio between the lengths of the Bi–S bond to the entire length of each molecule: *L*_*Bi*–*S*_/*L*_*Bi*–*SC*_*n*__. Then:
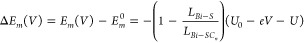
3Using reasonable values for the coupling constants of Γ_*L*_ = Γ_*R*_ = 5*meV*^47^ and the known values of *E*_*m*_^0^, allow us to plug for each value of *V*, Δ*E*_*m*_(*V*) into eqs 1 and 2 to calculate the I–V curves.

The results of this calculation for all chain lengths are plotted in Figure 4a and compared to the experimental I–V curves. The calculated RR values plotted as a function of applied bias are shown in Figure 4b. All calculated results agree with the magnitude of experimental rectification and its dependency on chain length. To emphasize this point Figure 4c compares the calculated and experimental RR values for all chains at ±1.0 V. The apparent excellent agreement in this plot is a major finding of this research and implies several important points: (i) Since the HOMO level in these junctions is localized on the thiol, the agreement with the model implies both the mitigation of Fermi level pinning and the effect of MIGS in these junctions, resulting from the attachment the molecules to a Bi lead. A similar conclusion was made in our previous thermovoltage measurements of the same junctions. (ii) The experimentally observed behavior of the RR as a function of molecular length can only be explained by considering the bias-dependent inner-potential within the Bi lead. In its absence, rectification should have been saturated with increasing molecular length as the ratio (in eq 3) *L*_*Bi*–*S*_/*L*_*Bi*–*SC*_*n*__ becomes smaller. Since the observed behavior is quite the opposite, for example the RR of SC_18_ at ±1.0 V is two times larger than of SC_16_ instead of being roughly the same, it infers that the effect of length on the magnitude of rectification comes from its effect on the induced potential within the Bi (Figures 3a and 3b). (iii) Considering the initial, −1.8 eV, position of the HOMO level (prior to the shift by U_0_) and using the same estimated coupling to the leads (5mev), the magnitude of rectification at ±1.0 V, should have been smaller than observed. This demonstrates another important advantage of using a semimetal (in this case Bi) as one of the leads. It allows tuning of molecular levels, by judiciously using the “knob” provided by the change in work function induced by the dipoles of the molecules. Such a capability can be used to enhance rectification by pushing, say the HOMO, even closer to the Fermi level by using conjugated molecules with the right average dipole in a monolayer. (iv) Recent studies demonstrating rectification based on the general model depicted in Figure 1a, suggest that the origin of Δ*E*_*m*_ is not in a potential divider across the molecules but a result of the Stark effect under the high electric field experienced by the molecules.^17,33,41^ Based on the Stark effect, since in the junctions studied here the electric field under a certain bias (*V*/*L*_*SC*_*n*__) progressively becomes smaller for longer molecules, rectification should have been less efficient for longer molecules. As such a trend is not observed, the Stark effect in these junctions can most likely be ruled out. (v) Since the work function of Au is ∼1 eV higher than of Bi, when a direct contact between these two materials is established a built-in potential of ∼1 eV within the Bi is created. We find that the resistance of such contacts (Bi evaporated directly on Au) is <1Ω, which proves that indeed rectification is not due to the built-in potential but due to the potential divider mechanism operating on the effective potential across the molecules.

**Figure 4:**
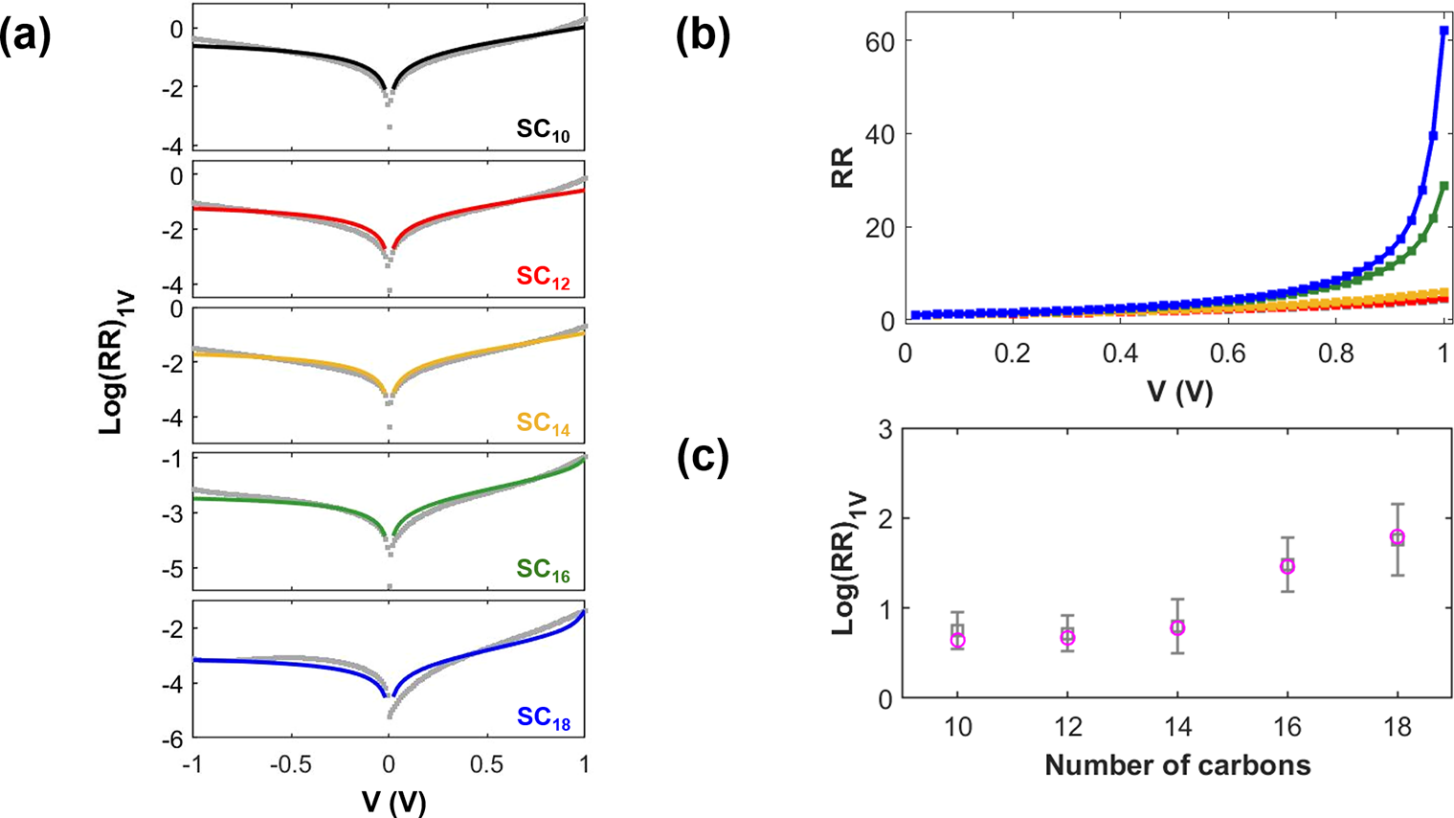
(a) Calculated I–V (log-scale) curves (colored lines) fitted to the averaged experimental data (gray squares). (b) Calculated RR vs applied bias. (c) log-normal-averaged experimental RR values at 1.0 V compared to the calculated values (magenta circles).

The rectification method presented here is not specific to the molecules used for its demonstration and can be expanded to other types of molecules, in particular conjugated ones. Then, the combined effect of initial resonance levels that are closer to the Fermi level, which can be shifted to be even closer by the appropriate molecular dipoles, along with the breaking of symmetry under bias provided by the semimetal, could potentially result in molecular rectifiers that are more relevant for technological applications. In terms of fundamental research, the above results suggest that the use of semimetals in molecular junctions, that is currently a totally unexplored territory, deserves further study. In addition, the capability presented here opens further options in junctions based on ’classical’ voltage-divider rectifiers such as ferrocenyl-alkanethiols, as then there are two molecular levels which response to the applied bias, separated by a spacer. In the SI we present an initial discussion on such a system.

## Supporting Information Available

Characterization of the Bi/monolayer interface by XPS, UPS measurements to determine the change in work function, contact angle measurements of the monolayers, measurements and their interpretation of the rectification in junctions with ferrocenyl-alkanethiols, and detailed calculations of the built-in potential within the semimetal with and without voltage bias (PDF)
