# Supplementary material for: Efficient Molecular Rectification in Metal–Molecules–Semimetal Junctions
Source: J Phys Chem Lett. 2024 Oct 15;15(42):10602–8. doi: 10.1021/acs.jpclett.4c02900 (PMC11514003; doi:10.1021/acs.jpclett.4c02900)
Supplement: Supplementary file 1 — jz4c02900_si_001.pdf [file jz4c02900_si_001.pdf]

Supporting Information

Efficient Molecular Rectification in Metal-Molecules-Semimetal Junctions

Shachar Shmueli, Mor Cohen Jungerman, Yoram Selzer*

[1]School of Chemistry, Tel Aviv University, Tel Aviv 69978, Israel.

[2]The Tel Aviv Center for Nanoscience and Nanotechnology, Tel Aviv 69978, Israel.

XPS measurements

The necessary conditions to strip-off the native oxide of Bi during the assembly process were determined by XPS measurements. The first attempt for this purpose is to use the peak of Bi-S bonds (158.6eV) in the Bi_{4f} regime, which is typically used in various synthesis procedures[1,2]. However, when trying to characterize the assembly of thiols on an oxide covered Bi film, the sole use of this peak becomes somewhat cumbersome due to its proximity to that of Bi-O (158.9eV). This point is demonstrated in Fig. 1Sa, which compares between the spectrum of a bare Bi film (covered with native oxide) and that of a similar film after SC_{14} assembly. While the peak of Bi (156.8eV) in both spectra is identical, the change between Bi-O to Bi-S is almost within the error of the measurement (0.05eV) indicated by the blue margin around the lines. We therefore believe that better analysis in this case should also be based on the S_{2s} regime of the spectra (Fig. 1Sb). Here, the results clearly show that assembly concentration of 100mM is needed for alkanethiols to form an interface with Bi-S bonds without any underlying oxide. This concentration has been used for the assembly of all molecules.

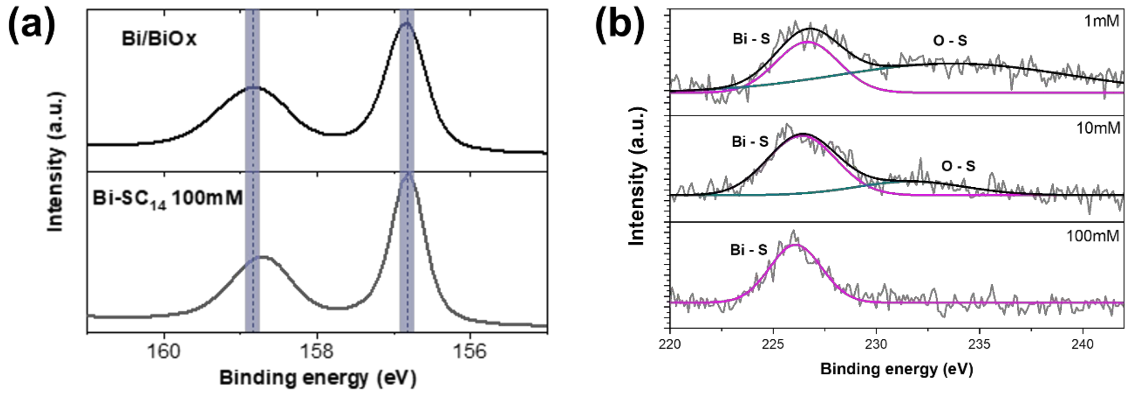

Figure 1S. (a) XPS Spectrum of a Bi film with its native oxide (upper panel) and of a similar film after assembly of SC_{14} from a 100mM solution (lower panel). Both spectra are in the Bi_{4f} regime. (b) XPS of SC_{14} monolayers on Bi

assembled from deoxygenated ethanol solution of the indicated concentrations of the alkanethiol measured in the S_{2s} regime.

Contact angle (CA) measurements

Similarly to the behavior of alkanethiols on metals such as Au and Ag[3] contact angle measurements of the used monolayers on Bi suggest that only monolayers of n≥10, i.e. with CA>100^0 can be regarded as well packed and ordered layers.

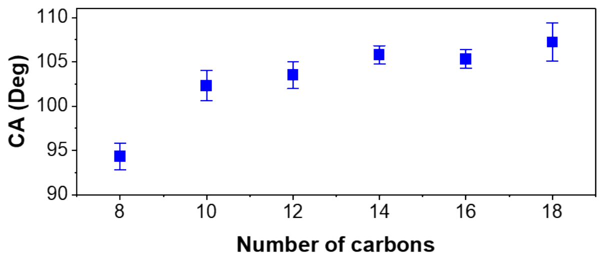

Figure 2S. Contact angles of water on alkanethiol monolayers on Bi.

UPS measurements

UPS spectra were obtained with 21.2 eV He (I) excitation (Omicron VUV Lamp HIS 13) and pass energy of 5 eV. For all UPS analyses, a 5 V bias was applied to improve the transmission of low kinetic energy (KE) electrons and to improve the determination of the energy edge in this regime. Between 22-30 Separate UPS spectra and XPS spectra were measured for a sputter-etched, atomically clean Au sample on each day of analysis before characterization of the monolayer-modified samples to ensure that instrument parameters were the same as for all previous studies. This process ensured that the low-KE edge, relative intensities of the low and high KE peaks, and the intensity of photoemission at the Fermi edge relative to the lowest KE region were consistent for all samples. All spectral features for identically modified samples appeared at energies reproducible to within ±0.05 eV.

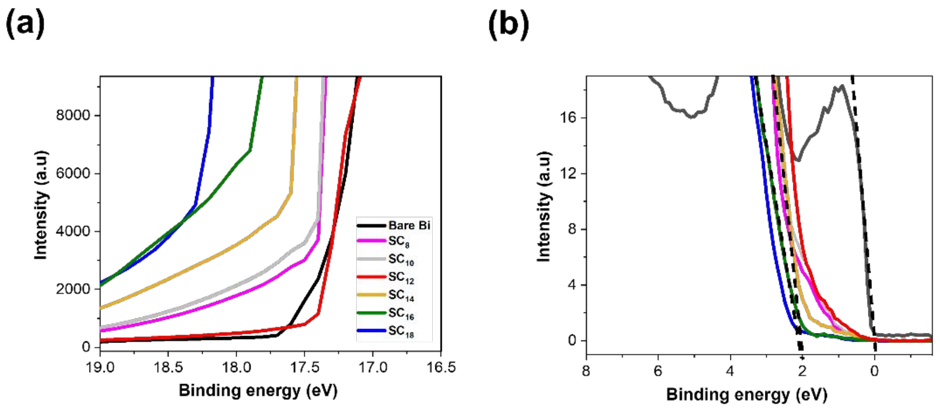

Figure 3S. (a) The change in work function was determined by the change in the photoemission cutoff caused by the different layers to the value obtained for an oxide free Bi surface. In all measurements the signal for oxide free Bi was collected from a Bi surface cleaned in-situ by sputtering. (b) In the photoemission onset all layers show a similar onset value attributed to the location of the HOMO level.

The values of ΔWF extracted from Fig. 3S are plotted in Fig. 3a in the main text and as described were used for the quantitative analysis of the rectification behavior. The sign of ΔWF is similar to that in previous reports of alkanethiols of Au[4-6]. In molecular terms this implies in both cases the effective R^+-S^- dipole (where $R=C_nH_{2n+1}$) must be larger than that of Bi^+-S^- and Au^+-S^-. There are, however, two notable differences between the two surfaces:

(a) While with alkanethiols on Au[5], ΔWF changes linearly with *n*, on Bi the behavior is linear only for *n*≥12. With shorter chains (*n*=8,10) ΔWF~0, essentially as for *n*=12.

(b) For alkanethiols on Au, the slope of ΔWF as function of *n* is ~10mV/CH_2 unit[5], while for Bi, in the linear regime, the slope is ~100mV/CH_2 unit.

The difference in the slope of ΔWF as a function of *n* is a result of the smaller dielectric constant of Bi, which causes less screening at the interface. The non-linear behavior is unique and most likely results from the properties of Bi, being a semimetal. The molecular dipole at the interface defining ΔWF can be modeled as[6]:

$$\Delta WF(eV) = eD_{\perp,total} = -Ne\left[\frac{D_{\perp,SAM}}{\varepsilon_0\varepsilon_{SAM}} + \frac{D_{Bi-S}}{\varepsilon_0\varepsilon_{BiS}}\right] \quad \text{(S1)}$$

where N is the density of molecules, $D_{\perp,SAM}$ is the R^+-S^- dipole projected on the normal to the surface, D_{Bi-S} is the Bi^+-S^- dipole, ε_0 is the permittivity of vacuum and ε_{SAM} and ε_{BiS} are the dielectric constant of the alkane chains and the Bi-S bond, respectively.

For n=12, ΔWF=0 and therefore to a first approximation at this point the two dipole contributions in equation S1 are equal in their absolute values, but opposite in sign and therefore cancel their overall contribution to the change in work function.

Extrapolation of the linear regime to n=0, results in:

$$\text{(S2)} \qquad \Delta WF(eV) = eD_{\perp,total} = -Ne\left[\frac{D_{Bi-S}}{\varepsilon_0 \varepsilon_{BiS}}\right] \cong 0.8eV$$

The corresponding values with Au and Ag, are for example, 0.49eV and 0.48eV, respectively[6]. The larger value with Bi can again be explained by less screening within the semimetal. However, the results suggest that the above extrapolation is not justified and the change in work function with n becomes highly non-linear and essentially nullified as the length of the alkane moiety becomes shorter, making the bond-dipole of Bi-S more dominant. The analysis of this behavior is beyond the scope of this paper and deserves additional measurements such as angle resolved photoemission spectroscopy to probe possible changes in the band structure at the interface[7,8].

Rectification in junctions with monolayers of Ferrocenyl-Alkanethiol (HS-(CH_2)$_{11}$-Fc).

Rectification in junctions with Ferrocenyl-Alkanethiol monolayers has been studied and analyzed extensively using the potential divider mechanism[9,10]. The HOMO and HOMO-1 of these monolayers reside on the Ferrocenyl and thiol end-groups, respectively. The analysis of their rectification typically considers only the former level, since no rectification is observed in junctions with alkanethiols and therefore no appreciable shift with bias of the HOMO-1 level has been assumed to occur.

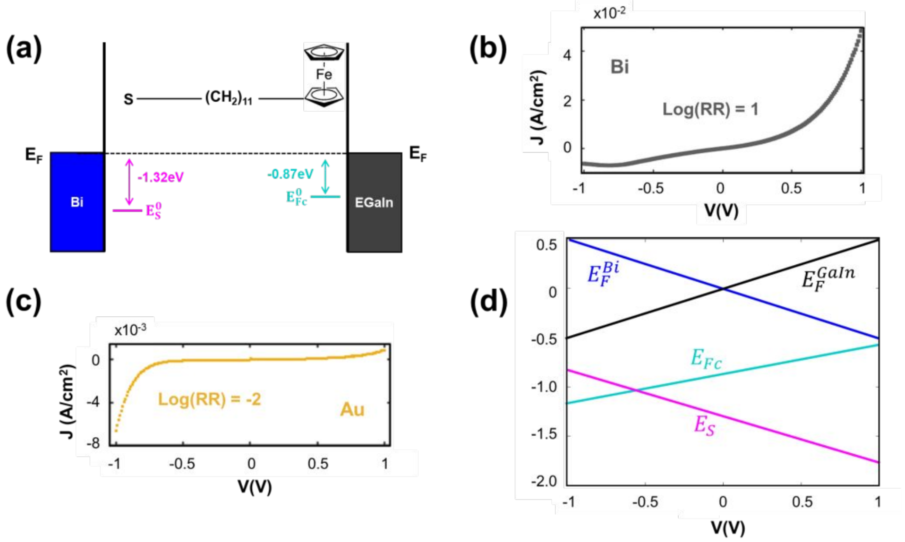

Figure 4S. (a) Level alignment of Ferrocenyl-Alkanethiol monolayer determined by UPS. (b) and (c) I-V curves of Ferrocenyl-Alkanethiol junctions with Bi and Au leads, respectively. (d) A plot showing how the energy of all involved levels is changing with bias. E_F^{Bi}, E_F^{GaIn}, E_{Fc} and E_S are the energies of the Fermi levels of the Bi and EGaIn leads and of the HOMO and HOMO-1 levels, respectively.

As rectification in this paper clearly demonstrates that the energy of a level localized on the thiol end connected to Bi can shift with bias, it would be interesting to examine the rectification of a junction with Ferrocenyl-Alkanethiol assembled on Bi. For this purpose, we have measured junctions with HS-(CH_2)$_{11}$-Fc. The long alkane chain ensures that the monolayers are well ordered and that the two involved electronic levels on both ends are well decoupled. This makes, to a first approximation, quantitative analysis of the rectification very simple. Following the model in the main text, we assume an *independent* Breit–Wigner resonance transmission behavior of both levels. For simplicity, considering their localized nature[10], the HOMO on the Ferrocenyl is assumed to be coupled solely to the EGaIn lead, and the HOMO-1 level on the thiol solely to the Bi. From UPS measurements of the monolayer on Bi the initial energy positions (before any bias) of the

HOMO and HOMO-1 are E_{Fc}^{0}=-0.87eV and E_{S}^{0}=-1.32eV below the Fermi level, respectively (Fig. 4Sa). Coincidently, the monolayer does not change the work function of Bi (ΔWF=0), which makes the I-V calculation more trivial. A self-consistent calculation of the potential drop at each bias value is not necessary. Typical I-V behavior of Bi- HS-$(CH_2)_{11}$-Fc-EGaIn and Au- HS-$(CH_2)_{11}$-Fc-EGaIn junctions are shown in Fig. 4Sb and Fig. 4Sc, respectively. The bias direction of rectification as well as magnitude of RR~100 for the junction with the Au lead is like previous results[11]. In comparison, with the Bi lead, the bias direction of rectification is reversed, and its magnitude is ten-fold smaller (RR~10). The I-V behavior of both types of junctions based on the simulations is depicted in Fig. 5S. We find good agreement both in rectification sign and magnitude. In the calculation, we assume an order of magnitude better coupling of the HOMO-1 level (on the thiol) to the Bi than of the HOMO level (on the Ferrocenyl) to the EGaIn, due to the oxide layer on the latter and the lack of covalent bond to this lead. The agreement between the model and experiment can be understood by examining Fig. 4Sd, which shows the shift of the involved levels with applied bias. In the case of junctions with Bi lead, both levels response and shift with bias and move closer to the window between the two Fermi levels of the leads: the HOMO at negative bias and the HOMO-1 at positive bias. As a result, the magnitude of rectification is modest. In contrast, with a Au lead, the shift of the HOMO-1 level is not taking place and the RR is larger. In both cases the sign of rectification is a result of a combined effect involving the shift of levels and the magnitude of their coupling to the leads.

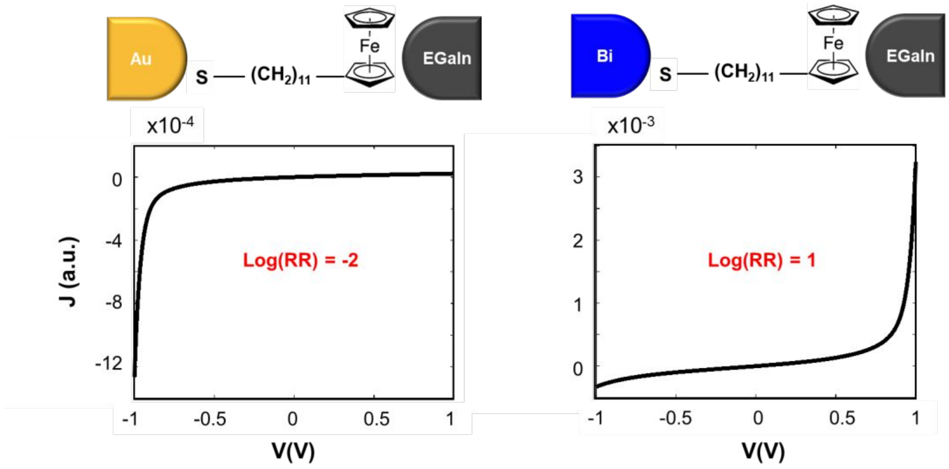

Figure 5S. Simulated I-V curves of the indicated junctions. Current is in arbitrary units. See text for details.

Calculation of the space-charge region in Bi

The calculation is based on a one-dimensional Poisson's equation for a one-electron potential energy u given by:

(S1) $$\frac{d^2u}{dx^2} = \frac{4\pi e^2}{\epsilon_{Bi}}[n_e(x) - n_h(x)]$$

where ϵ_{Bi} is the permittivity of Bi ($\epsilon_{Bi} = 100$). The quantities $n_e(x)$ and $n_h(x)$ are the particle densities of electrons and holes, respectively and we assume that Bi extends from $x = 0$ to $x = \infty$.

The boundary conditions of equation S1 are:

(S2a) $$\lim_{x\to\infty} u(x) = 0$$

(S2b) $$\lim_{x\to\infty} du/dx = 0$$

At zero temperature the particles densities are given by the Fermi-Thomas model to be:

(S3a) $$n_e(x) = n_0\left[1 - \frac{u}{\xi_e}\right]^{\frac{3}{2}}\theta(\xi_e - u)$$

(S3b) $$n_h(x) = n_0\left[1 - \frac{u}{\xi_h}\right]^{\frac{3}{2}}\theta(|\xi_h| - u)$$

where n_0 is the bulk density of electrons and holes (3.5×10^{17} cm^{-3}) and ξ_e (=22meV) and ξ_h (=12meV) are respectively their Fermi energies.

Using

(S4) $$\theta(x) = 1 \; ; x > 0$$

$$=0 \; ; \; x < 0$$

equation (S1) can be reduced to:

(S5a) $$x = \left(\frac{5\epsilon_{Bi}}{16\pi n_0 e^2}\right)^{1/2} \int_0^{u(x)} du/D(u)$$

(S5b) $$D(U) = \left[\zeta_h\left[1 + \frac{U}{\zeta_h}\right]^{\frac{5}{2}}\theta\left(1 + \frac{U}{\zeta_h}\right) + \zeta_e\left[1 - \frac{U}{\zeta_e}\right]^{\frac{5}{2}}\theta\left(1 - \frac{U}{\zeta_e}\right) - (\zeta_h + \zeta_e)\right]^{\frac{1}{2}}$$

The quantity u(0) is calculated assuming that $\Delta\varphi$, the difference between the work functions of the metal (EGaIn, 4.15eV) and Bi (4.3+ΔWF) is divided between a molecular layer of thickness x_{mol} from $x = -x_{mol}$ to $x = 0$ and the semi-infinite Bi. The boundary conditions at the molecular layer/Bi interface are:

(S6a) $$u(x\rightarrow 0_{mol}) = u(x\rightarrow 0_{Bi})$$

(S6b) $$\epsilon_{mol}u'(x\rightarrow 0_{mol}) = \epsilon_{Bi}u'(x\rightarrow 0_{Bi}) \text{ (using } \epsilon_L = 2\text{).}$$

(S6c) $$u(x) = F(x + x_{mol}), \quad -x_{mol} \leq x \leq 0$$

(S6d) $$\Delta\varphi = -u(0) + Fx_{mol}$$

where F is the field across the molecular layer.

By taking the derivative of equations S5a and S6c, we can eliminate F and obtain the following set of equations:

(S7a) $$U \equiv u(0) = -\Delta\varphi + \epsilon_R u'(0)x_{mol}/\epsilon_L$$

(S7b) $$u'(0) = -\left(\frac{16\pi n_0 e^2}{5\epsilon_R}\right)^{\frac{1}{2}} D(U)$$

which are then solved iteratively.

Calculation of the space-charge region under bias

When a potential bias is applied, equations S7 are modified to be:

(S8a) $$U \equiv u(0) = -\Delta\varphi - eV + \epsilon_R u'(0)x_{mol}/\epsilon_L$$

(S8b) $$u'(0) = -(signV)\left(\frac{16\pi n_0 e^2}{5\epsilon_R}\right)^{\frac{1}{2}} D(U)$$

which can also be solved iteratively to calculate U.

The formed built in potential is then:

(S9a) $$u(x) = Uexp(-\kappa_D x)$$

(S9b) $$\kappa_D^2 = \frac{6\pi n_0 e^2}{\epsilon_R}\left[\frac{1}{\zeta_e} + \frac{1}{|\zeta_h|}\right]$$

Plots of S9 at zero bias for the different chains are shown in Figure 3b in the main text.

References

1. Sambathkumar, C.; Manirathinam, V.; Manikandan, A.; Krishna Kumar, M.; Sudhahar, S.; Devendran, P. *J Mater Sci: Mater Electron* **2021**, 32, 20827.

2. Wang, S.; Li, W.; Song, H.; Mao, C.; Zhang, Z.; Peng, H.; Li, G. *Inorg. Chem. Front.* **2019**, 6, 1275

3. Atre, S.V.; Liedberg, B.; Allara, D.L. *Langmuir* **1995**, *11*, 3882–3893.

4. Alloway, D. M.; Hofmann, M.; Smith, D. L.; Gruhn, N. E.; Graham, A. L.; Colorado, R.; Wysocki, V. H.; Lee, T. R.; Lee, P. A.; Armstrong, N. R. *J. Phys. Chem. B* **2003**, *107,* 11690-11699.

5. Evans, S. D. *Chem. Phys. Lett.* **1990**, 170, 462

6. Alloway, D. M.; Graham, A. L.; Yang, X.; Mudalige, A.; Colorado, R.; Wysocki, V. H.; Pemberton, J. E.; Lee, T. R.; Wysocki, R. J.; Armstrong, N. R. *J. Phys. Chem. C* **2009,** *113,* 20328–20334.

7. Ito, S.; Feng, B.; Arita, M.; Someya, T.; Chen, W. C. Takayama, A.; Iimori, T.; Namatame, H.; Taniguchi, M.; Cheng, C. M.; Tang, S. J. Komori, F.; Matsuda, I. Phys. Rev. B. **2018,** 97, 155423.

8. Matetskiya, A. V.; Bondarenkoa, L. V.; Tupchayaa, A. Y.; Gruzneva, D. V.; Eremeevb, S. V.; Zotova, A. V.; Sara,nina A. A. App. Surf. Sci. **2017**, 406 122–127.

9. Van Nguyen, Q. *J. Phys. Chem. C.* **2022**, 126, 6405.

10. Yuan, L.; Nerngchamnong, N.; Cao, L.; Hamoudi, H.; del Barco, E.; Roemer, M.; Sriramula, R. K.; Thompson, D.; Nijhuis, C. A. Nat. Comm. 2015, 6, 6324.

11. Reus, W. F.; Thuo, M. M.; Shapiro, N. D.; Nijhuis, C. A.; Whitesides, G. M. ACS Nano 2012, 6, 4806.
